# Apelin-13 Pretreatment Attenuates Age-Associated Renal Fibrotic Remodeling Following I/R Injury

**DOI:** 10.3390/biomedicines14092033

**Published:** 2026-09-10

**Authors:** Won-Seok Oh, Sang Gon Lee, Hyun Tae Kim, Ji-Hyun Moon, Ah-La Choi, Seung Yun Han, Do Kyung Kim, Nam Seob Lee, Young Gil Jeong, Geum-Lan Hong

**Affiliations:** 1 Department of Veterinary Anatomy, College of Veterinary Medicine, Chungnam National University, Daejeon 34134, Republic of Korea; eric000705@naver.com (W.-S.O.); gon@o.cnu.ac.kr (S.G.L.); 2 Honam Regional Center, Korea Basic Science Institute (KBSI), Gwangju 61751, Republic of Korea; hyuntae90@kbsi.re.kr; 3 Department of Anatomy, College of Medicine, Konyang University, Daejeon 35365, Republic of Korea; smile8061@gmail.com (J.-H.M.); free136@konyang.ac.kr (A.-L.C.); dokyung@konyang.ac.kr (D.K.K.); nslee@konyang.ac.kr (N.S.L.); ygjeong@konyang.ac.kr (Y.G.J.); 4 Medical Department, Cheongju Women’s Correctional Institution, Ministry of Justice, Cheongju 28634, Republic of Korea; jjzzy9448@gmail.com

**Keywords:** aging, apelin signaling, acute kidney injury, renal fibrosis, TGF-β1/SMAD3 signaling

## Abstract

**Background/Objectives**: Aging is a major risk factor that exacerbates acute kidney injury (AKI) and subsequent renal fibrosis. However, age-related factors contributing to post-AKI fibrotic remodeling remain underexplored. This study aimed to investigate the protective potential of apelin, an endogenous peptide, in mitigating post-AKI renal fibrosis in aged mice. **Methods**: A mouse model of AKI was established in 8-week-old and 19-month-old male C57BL/6 mice by clamping the left renal artery for 40 min, followed by reperfusion and euthanasia 2 weeks post-ischemia. To evaluate the protective effect of apelin pretreatment, the 19m-APLN group received intraperitoneal apelin administration (25 μg/kg/day) for 2 weeks prior to AKI induction. The effects of apelin on renal fibrosis were evaluated using renal function tests (blood urea nitrogen, serum creatinine levels), histological analysis, and Western blotting. **Results**: The 19m-AKI group exhibited more severe renal damage and interstitial fibrosis, along with a marked reduction in apelin and APLNR expression compared to the 8wk-AKI group. Apelin-13 pretreatment attenuated renal injury and fibrotic remodeling in aged mice, with reduced collagen deposition accompanied by lower pro-TGF-β1 expression and SMAD3 phosphorylation. **Conclusions**: These results highlight the protective role of apelin against age-related exacerbation of post-AKI renal fibrosis, suggesting that prophylactic Apelin-13 administration may attenuate post-I/R renal injury and fibrotic remodeling in aged kidneys.

## 1. Introduction

As the global population ages, the incidence of acute kidney injury (AKI) and its progression to chronic kidney disease (CKD) have emerged as major clinical burdens [1,2]. AKI is characterized by a rapid decline in the glomerular filtration rate over a period of hours to days, leading to the accumulation of nitrogen wastes and diverse clinical manifestations [3]. Although AKI and CKD were historically considered distinct diseases [4], recent epidemiological studies indicate that they form an interconnected disease spectrum, wherein unresolved acute tubular injury frequently triggers progressive tubulointerstitial fibrosis [5,6,7]. Consequently, recent studies have focused on elucidating the molecular mechanisms underlying post-AKI renal fibrosis for developing targeted therapeutic strategies [8].

In older individuals, AKI is challenging to treat owing to their heightened susceptibility to renal insults and increased severity of kidney injury [9,10]. Unlike young kidneys capable of adaptive repair, aged kidneys suffer from microvascular rarefaction, elevated oxidative stress, and defective tubular regeneration, which collectively bias the post-injury response toward maladaptive remodeling and persistent fibrogenesis [11,12,13]. The irreversible loss of nephrons following AKI can accelerate the renal function decline and promote progression to chronic kidney failure [14]. Although age-related alterations in cellular signaling pathways have been implicated in the pathogenesis of AKI in the elderly [15,16,17], the specific molecular events and pathways that exacerbate post-AKI renal fibrosis in the aged kidney remain poorly understood.

The Apelinergic system, an endocrine system in the body comprising two endogenous ligands, Apelin (encoded by the *Apln* gene), APELA (encoded by the *Apela* gene), and the G-protein-coupled Apelin receptor (APLNR; encoded by the *Aplnr* gene), functions as a key regulator of cardiovascular and renal homeostasis [18,19]. Expressed abundantly in vascular endothelial cells and renal tubular epithelium, apelin exerts anti-inflammatory, pro-angiogenic, and anti-fibrotic effects across multiple organ systems. Specifically, apelin administration attenuates myocardial remodeling and cardiac fibrosis [20], mitigates pulmonary vascular remodeling and lung fibrosis [21], and inhibits hepatic stellate cell activation in liver fibrosis [22]. In the kidney, apelin plays an essential protective role by preserving endothelial barrier integrity, enhancing microvascular perfusion, and mitigating acute tubular necrosis during ischemia–reperfusion injury [23,24]. Previous studies have reported anti-fibrotic effects of apelin associated with modulation of TGF-β1/SMAD2/3 signaling and fibrosis-related molecular changes [25,26].

Importantly, an aging-associated decline in endogenous apelin and APLNR expression has been observed across multiple tissues, which may compromise basal repair mechanisms and exacerbate susceptibility to post-injury fibrogenesis [27,28]. Despite these recognized renoprotective actions and the known age-related deficit in apelinergic signaling, the preventive effect of exogenous apelin treatment on post-AKI fibrotic progression remains unexplored. Therefore, this study aimed to evaluate whether apelin pretreatment exerts a protective effect against post-AKI renal damage and explore the associated molecular pathways. Understanding these mechanisms is essential for developing targeted preventive strategies to mitigate renal injury and improve outcomes in older populations. We hypothesized that Apelin-13 (the predominant biologically active isoform) pretreatment would attenuate renal injury and fibrotic remodeling in aged mice, accompanied by reduced TGF-β1/SMAD3 signaling.

## 2. Materials and Methods

### 2.1. Animals and Treatments

All experimental procedures followed the Guide for the Care and Use of Laboratory Animals (National Institutes of Health, Bethesda, MD, USA) and were approved by the Animal Ethics Committee of Konyang University (Daejeon, Republic of Korea; P-23-15-E-01), and were reported in accordance with the Animal Research: Reporting of In Vivo Experiments (ARRIVE) guidelines (https://arriveguidelines.org) [29]. Thirty male C57BL/6 mice obtained from the Animal Facility of Aging Science, Korea Basic Science Institute (KBSI), Gwangju Center (Gwangju, Republic of Korea), were divided into five experimental groups (*n* = 6 per group): 8-week-old control (8wk-CON), 8-week-old I/R-induced AKI (8wk-AKI), 19-month-old control (19m-CON), 19-month-old I/R-induced AKI (19m-AKI), and 19-month-old I/R-induced AKI treated with apelin (19m-APLN). All mice were kept in a specific pathogen-free facility under controlled environmental conditions (temperature of 20–24 °C, relative humidity of 40–60%, and a 12 h light/dark cycle) and provided with ad libitum access to standard laboratory rodent diet and tap water. They were acclimatized for 1 week prior to the start of the experiments. Six 19m-APLN mice received intraperitoneal injections of synthetic Apelin-13 (Apelin-13; MedChemExpress, Monmouth Junction, NJ, USA, Cat# HY-P1944; 25 μg/kg/day) for 2 weeks before the induction of ischemia–reperfusion (I/R)-induced AKI. Apelin was dissolved in sterile saline (0.9% NaCl) to a concentration of 2.5 µg/mL and administered at 10 µL/g body weight.

### 2.2. I/R-Induced Acute Kidney Injury

Renal I/R injury was induced under isoflurane (Hana Pharm, Seoul, Republic of Korea) anesthesia (2% induction, 1.5% maintenance). Body temperature was maintained using a heating pad and vital signs, including heart and respiratory rates, were continuously monitored throughout the procedure. After placing the mouse in dorsal recumbency, the abdominal area was disinfected with iodine solution followed by 70% ethanol. A midline abdominal incision was made to expose the kidneys. The left renal artery was carefully isolated and occluded using a nontraumatic microvascular clamp. The duration of ischemia was set to 40 min. After the ischemic period, the clamp was removed to allow kidney reperfusion. The abdominal incision was closed, and the mice were monitored until they fully recovered from the anesthesia. The 8wk-CON and 19m-CON groups served as age-matched intact controls and did not undergo sham surgery. After 2 weeks, all mice were euthanized with carbon dioxide (CO_2_) gas in accordance with established ethical guidelines. Following euthanasia, transcardiac perfusion was performed with 100 mL of ice-cold phosphate-buffered saline (PBS) to clear residual blood from the kidneys. The left kidneys were harvested from all experimental groups, corresponding to the ipsilateral kidney subjected to renal artery clamping in the I/R groups, and sectioned transversely into three equal portions. The middle part was fixed in 10% neutral buffered formalin (Sigma-Aldrich, St. Louis, MO, USA) for histological and immunohistochemical analyses, while the remaining parts were stored in RNAlater Stabilization Solution (Thermo Fisher Scientific, Waltham, MA, USA) for microarray analysis and fresh-frozen for Western blot analyses.

### 2.3. Measurement of Kidney Function

Blood samples were collected into serum separating tubes. The collected whole blood was allowed to clot at room temperature for 30 min, followed by centrifugation (1200 rpm, 15 min, 23 °C) to separate the serum. The levels of the kidney function markers, blood urea nitrogen (BUN) and serum creatinine (SCr), were measured using an automated dry chemistry analyzer (FUJIFILM DRI-CHEM 7000i; Fujifilm Corporation, Tokyo, Japan) according to the manufacturer’s instructions.

### 2.4. Microarray Analysis

The dissected kidney cortex samples from all four experimental groups (8wk-CON, 8wk-AKI, 19m-CON, and 19m-AKI; *n* = 2–3 per group) were subjected to transcriptomic profiling via Macrogen (Seoul, Republic of Korea). The data were summarized and normalized using the robust multi-array average (RMA) method implemented in Affymetrix Power Tools (APT, version 2.11.8, Thermo Fisher Scientific). Gene-level RMA analysis results were exported, and differentially expressed gene (DEG) analysis was performed to compare the experimental groups using an independent Student’s *t*-test and fold change (FC) analysis. Genes satisfying the criteria of |FC| > 2 and raw *p* < 0.1 were defined as candidate DEGs and visualized using a volcano plot. No multiple-testing adjustment was applied for DEG selection.

To further characterize transcriptional pathways associated with renal fibrotic remodeling following I/R injury, pre-ranked Gene Set Enrichment Analysis (GSEA) was performed against the standardized MSigDB Hallmark Epithelial–Mesenchymal Transition (EMT) gene set (M5930) with 1000 permutations. In addition, to track the expression trajectory of the apelinergic axis across aging and ischemic conditions, a heatmap of row-wise Z-scores was generated encompassing the core apelinergic components (*Apela*, *Apln*, and *Aplnr*) alongside their downstream signaling effectors and regulated target genes. Data processing, statistical computations, and visualizations were performed using Python (version 3.13) and GraphPad Prism (version 8.0; GraphPad Software, San Diego, CA, USA).

### 2.5. Histopathological Analysis

The fixed tissue was embedded in paraffin to create tissue blocks, and representative sections containing both the cortex and medulla were obtained from the mid-portion of the paraffin-embedded kidney blocks and transversely sectioned at a thickness of 5 µm using a rotary microtome. Slides containing these sections were deparaffinized with xylene, hydrated with ethanol, and stained with hematoxylin and eosin (H&E), periodic acid-Schiff (PAS), and Sirius Red.

For H&E staining, sections were stained with Mayer’s hematoxylin (Dako, Agilent Technologies, Santa Clara, CA, USA) for 70 s, and eosin (Labcore, Seoul, Republic of Korea) for 90 s. For PAS staining, sections were treated with 0.5% periodic acid (prepared in distilled water) for 5 min, incubated in Schiff’s solution (Sigma-Aldrich) for 30 min, and counterstained with Mayer’s hematoxylin. For Sirius Red staining, sections were stained for 15 min with Weigert’s iron hematoxylin, which was prepared by mixing 1% hematoxylin in 95% ethanol with an iron mordant solution (4% ferric chloride and 1% hydrochloric acid in distilled water, both from Sigma-Aldrich) at a 1:1 ratio. Subsequently, sections were incubated in Picro-Sirius Red solution (0.1% Sirius Red F3BA in saturated aqueous picric acid; Sigma-Aldrich) for 50 min and rinsed three times with acidified water (0.5% hydrochloric acid in distilled water). After staining, sections were dehydrated, cleared, and mounted with Canada balsam (Sigma-Aldrich).

To ensure objective evaluation, 10 non-overlapping fields from the renal cortex per specimen were randomly selected and analyzed in a double-blinded manner by two independent observers. The values measured from the 10 fields per mouse were averaged to represent each individual animal for subsequent statistical analysis (*n* = 6 per group).

The severity of renal damage was quantified using the Endothelial, Glomerular, Tubular, and Interstitial scoring system [30], with specific stains and criteria applied to each compartment. Tubular damage was evaluated on H&E-stained sections and graded from 0 to 4 based on the Endothelial, Glomerular, Tubular, and Interstitial criteria as follows: 0, no damage; 1, loss of brush border in <25% of tubular cells; 2, loss of brush border in >25% of cells with a thickened basal membrane; 3, presence of inflammation, cast formation, and necrosis in up to 60% of cells; and 4, necrosis in >60% of tubular cells. Glomerular damage was assessed on PAS-stained sections using a modified 0–4 scale to reflect ischemic progression: 0, normal glomerular architecture; 1, thickening of Bowman’s capsule; 2, retraction of the glomerular tuft; 3, glomerular fibrosis or significant matrix expansion; and 4, disruption or rupture of Bowman’s capsule (loss of the parietal epithelial layer). The extent of renal interstitial fibrosis was quantified using Sirius Red staining. The Sirius Red-positive collagen area (red staining) was measured using ImageJ software (ImageJ v1.46a; National Institutes of Health, Bethesda, MD, USA).

### 2.6. Immunohistochemistry

After the 5 μm sections were deparaffinized and hydrated as described in Section 2.5, immunohistochemistry was performed. The sections were boiled in antigen retrieval solution (sodium citrate buffer, pH 6.0; Sigma-Aldrich) to unmask the epitope. After cooling to room temperature, endogenous peroxidase activity was quenched with 3% H_2_O_2_ in 100% methanol, and sections were permeabilized with 0.5% Triton X-100 (Sigma-Aldrich) in PBS for 30 min at room temperature. Sections were then incubated with normal goat serum (Vector Laboratories, Newark, CA, USA) for 1 h to reduce nonspecific binding. After applying the primary antibody (anti-APLNR, 1:100, Thermo Fisher Scientific), sections were incubated at 4 °C overnight in a humidified chamber. The following day, the sections were incubated with secondary horseradish peroxidase-conjugated anti-rabbit IgG antibody (1:200, GenDEPOT, Katy, TX, USA) for 2 h at room temperature, followed by incubation with the VECTASTAIN ABC Kit (Vector Laboratories) for 30 min at room temperature to conjugate horseradish peroxidase. Antibody binding was then visualized using a Diaminobenzidine (DAB) Substrate Kit (Vector Laboratories) in a dark room. Once adequate color development was achieved, the reaction was stopped by rinsing with triple-distilled water. After counterstaining with Mayer’s hematoxylin, the sections were dehydrated, cleared, and mounted with Canada balsam.

Digital images were captured using a digital slide scanner (KF-FL-005, KFBIO, Ningbo, China). To ensure objectivity and representative sampling, 10 fields were randomly selected and captured in a blinded manner. The mean values obtained from these 10 regions were used for subsequent statistical analysis. For the quantification of protein expression, immunohistochemically stained sections were analyzed using Fiji software (version 1.51h; an open-source distribution of ImageJ). To isolate the DAB-specific signal, the Colour Deconvolution plugin with H DAB vectors was applied. The resulting DAB channel was converted into an 8-bit grayscale image. The Mean Gray Value was measured to evaluate the overall protein expression levels. The relative intensity of the protein expression was quantified by calculating the Optical Density (OD) using the following formula: OD = log10(255/Mean Gray Value).

### 2.7. Western Blot

For protein extraction, tissues were homogenized using a bead mill homogenizer (Omni International, Kennesaw, GA, USA). Each tissue sample was placed in a 1.5 mL microcentrifuge tube with 500 μL of 1× radio-immunoprecipitation assay (RIPA) buffer and three beads, followed by homogenization. The 1× RIPA buffer was prepared by mixing 5 mL of 10× radioimmunoprecipitation assay buffer (Cell Signaling Technology, Danvers, MA, USA), 45 mL of distilled water, and one protease inhibitor cocktail tablet (cOmplete™, Roche Diagnostics, Basel, Switzerland) per 50 mL of solution. After homogenization, the tubes were cooled on ice to allow the foam to settle. The samples were then centrifuged at 12,000 rpm for 20 min at 4 °C using a high-speed refrigerated microcentrifuge. The supernatant was collected in a fresh tube. Protein concentrations were determined using the Pierce™ BCA Protein Assay Kit (Thermo Fisher Scientific). Finally, 5× sample buffer (SmartGene, Daejeon, Republic of Korea), 1× RIPA buffer, and the collected supernatants were mixed in an appropriate ratio and boiled at 95–100 °C for 10 min to prepare protein samples for Western blotting. Protein samples were separated on 8%, 10%, 12%, and 15% sodium dodecyl sulfate–polyacrylamide gels at 40 V and 400 mA. The size-fractionated proteins were transferred onto polyvinylidene fluoride membranes (Millipore, Billerica, MA, USA) at 40 V and 400 mA at 4 °C for 16.5 h. Following the transfer, membranes were sectioned prior to blocking incubation to allow simultaneous probing of different target proteins. (Consequently, full-length images of some blots with visible membrane edges are not available. Representative blots for each condition, with membrane edges shown where possible, are provided in the Supplementary Information.)

The membranes were then blocked with 5% skim milk in PBS containing 0.1% Tween-20 (PBS-T; Sigma-Aldrich) at room temperature for 2.5 h. The membranes were then incubated overnight at 4 °C with primary antibodies in 5% bovine serum albumin (Georgia Chem, Norcross, GA, USA) in PBS-T. The primary antibodies used were anti-β-Actin (1:1000, Abcam, Cambridge, UK), anti-KIM-1 (1:1000, MyBioSource, Vancouver, BC, Canada), anti-Apelin (1:1000, Boster Bio, Pleasanton, CA, USA), anti-APLNR (1:1000, Santa Cruz Biotechnology, Dallas, TX, USA), anti-TGF-β1 (detecting pro-TGF-β1, 1:1000, Abcam), anti-phospho-SMAD3 (1:1000, ABclonal Technology, Woburn, MA, USA), anti-SMAD3 (1:1000, Santa Cruz Biotechnology), anti-α-SMA (1:1000, Abcam), anti-N-cadherin (1:1000, Cell Signaling Technology). The following day, the membranes were incubated with horseradish peroxidase-conjugated anti-mouse and anti-rabbit IgG secondary antibodies (1:5000; GenDEPOT) in 5% skim milk with PBS-T at room temperature for 100 min. Protein signals were developed using Westglow Pico/Femto Chemiluminescent Substrate (Biomax, Guri, Republic of Korea) and captured on a WSE-6370 LuminoGraph III Lite (ATTO, Tokyo, Japan).

For TGF-β1, the approximately 45 kDa band corresponding to the precursor (pro-TGF-β1) form was used for quantitative analysis. To detect total SMAD3 on the same membrane, the membrane previously probed for Phospho-SMAD3 was stripped using Ultra-Fast Reprobe Solution (SmartGene) according to the manufacturer’s instructions. Following stripping, the membrane was re-blocked and incubated overnight at 4 °C with anti-SMAD3 primary antibody (1:1000; Santa Cruz Biotechnology), followed by secondary antibody incubation and ECL detection as described above. Densitometric quantification of protein bands was performed using CS Analyzer 4 (ATTO). Final data analysis and graph generation were performed using GraphPad Prism.

### 2.8. Statistics

Data are presented as mean ± standard deviation (SD). Normality and homogeneity of variance were assessed using the Shapiro–Wilk test and Levene’s test, respectively. As the assumptions for parametric analysis were satisfied, differences among groups were evaluated using one-way analysis of variance (ANOVA) followed by Tukey’s post hoc test for multiple comparisons. Statistical analyses were performed using GraphPad Prism and SigmaPlot (version 12.0; Grafiti LLC, Palo Alto, CA, USA) software. The threshold for statistical significance was set at *p* < 0.05; statistical significance is indicated as: *p* < 0.001 (***), *p* < 0.01 (**), and *p* < 0.05 (*).

## 3. Results

### 3.1. Aging Exacerbates I/R-Induced Acute Kidney Injury in Mice

**Figure 1:**
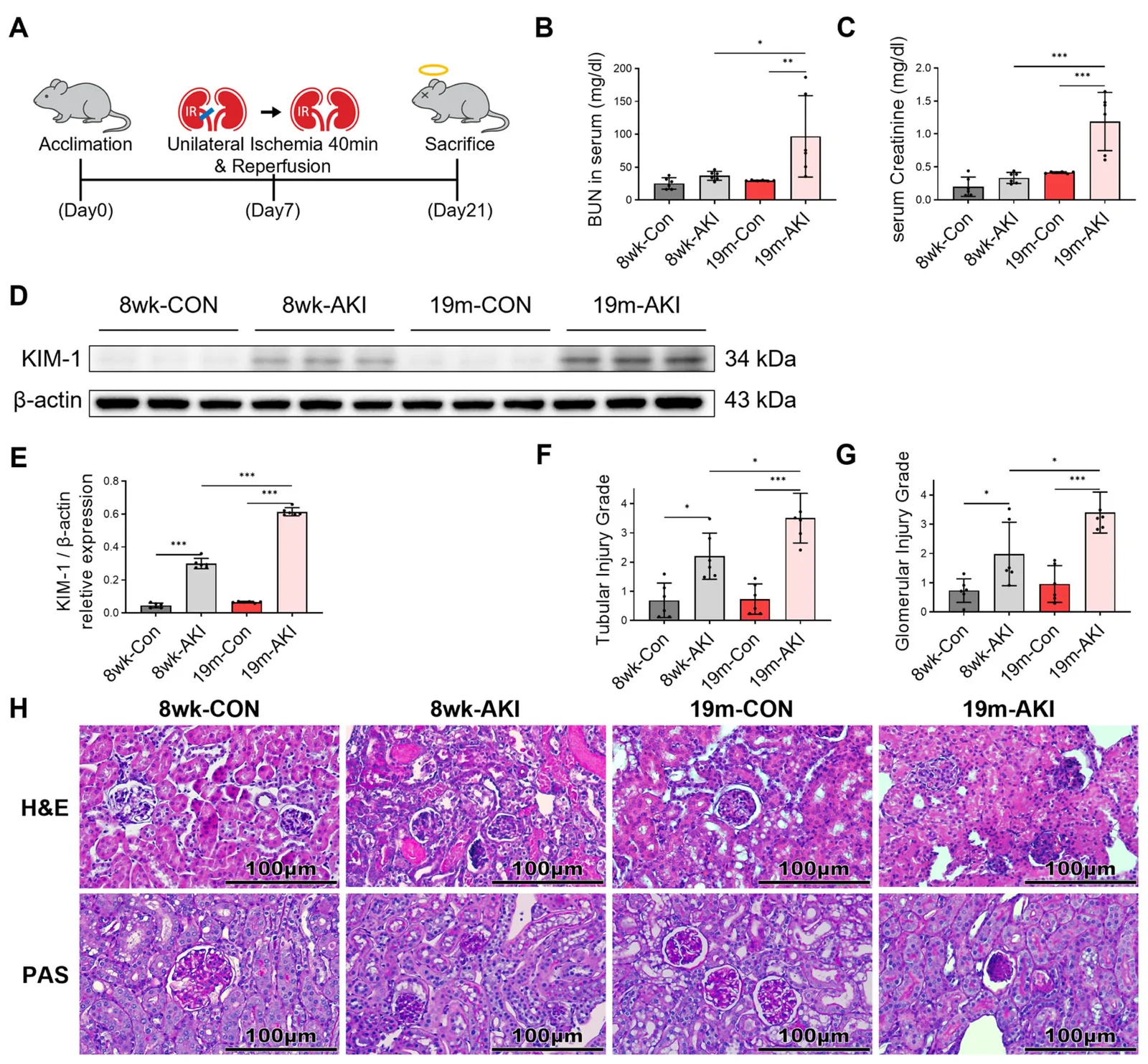
Exacerbation of ischemia–reperfusion (I/R)-induced renal injury in aged mice compared to young mice. (**A**) Schematic timeline of the unilateral renal I/R model. (**B**) Serum blood urea nitrogen (BUN) levels in each group (*n* = 6 mice per group). (**C**) Serum creatinine (SCr) levels in each group (*n* = 6 mice per group). (**D**) Representative Western blot analysis of kidney injury molecule-1 (KIM-1) expression in mouse kidneys, and (**E**) its quantification normalized to β-actin (*n* = 6 per group). (**F**) Evaluation of tubular injury grade and (**G**) glomerular injury grade (*n* = 6 mice per group). (**H**) Representative histological images of kidney sections stained with hematoxylin and eosin (H&E, top row) and periodic acid-Schiff (PAS, bottom row). Scale bar = 100 µm (400×). Data are expressed as mean ± SD. Statistical significance was analyzed using one-way analysis of variance (ANOVA) followed by Tukey’s post hoc test (*** *p* < 0.001, ** *p* < 0.01, and * *p* < 0.05).

To evaluate the impact of aging on acute kidney injury, a mouse model of unilateral renal I/R injury was established in 8-week-old and 19-month-old mice (Figure 1A). Biochemical analyses revealed significantly greater renal dysfunction in aged mice following I/R. No significant differences in BUN and SCr levels were observed between the control groups (8wk-CON vs. 19m-CON). However, serum BUN levels were significantly higher in the 19m-AKI group than in the 8wk-AKI group (Figure 1B). Similarly, SCr levels were significantly higher in the 19m-AKI group than in the 8wk-AKI group (Figure 1C). Although I/R injury significantly increased kidney injury molecule-1 (KIM-1), a well-established biomarker of renal tubular damage in young mice (8wk-CON vs. 8wk-AKI), its expression was further exacerbated in the 19m-AKI group (Figure 1D,E). Histopathological evaluation revealed normal glomerular and tubular structures in both control groups. However, the 8wk-AKI group exhibited moderate injury, including glomerular shrinkage, tubular dilation, loss of brush borders, and cytoplasmic vacuolization. These pathological changes were substantially exacerbated in the 19m-AKI group, which exhibited extensive tubular epithelial necrosis, cell detachment, and pronounced luminal dilatation (Figure 1F–H). Collectively, these results indicate that aging does not impair renal function under baseline conditions but markedly enhances the susceptibility to and severity of I/R-induced kidney injury.

### 3.2. Aging Aggravates Renal Fibrotic Remodeling Following I/R Injury

**Figure 2:**
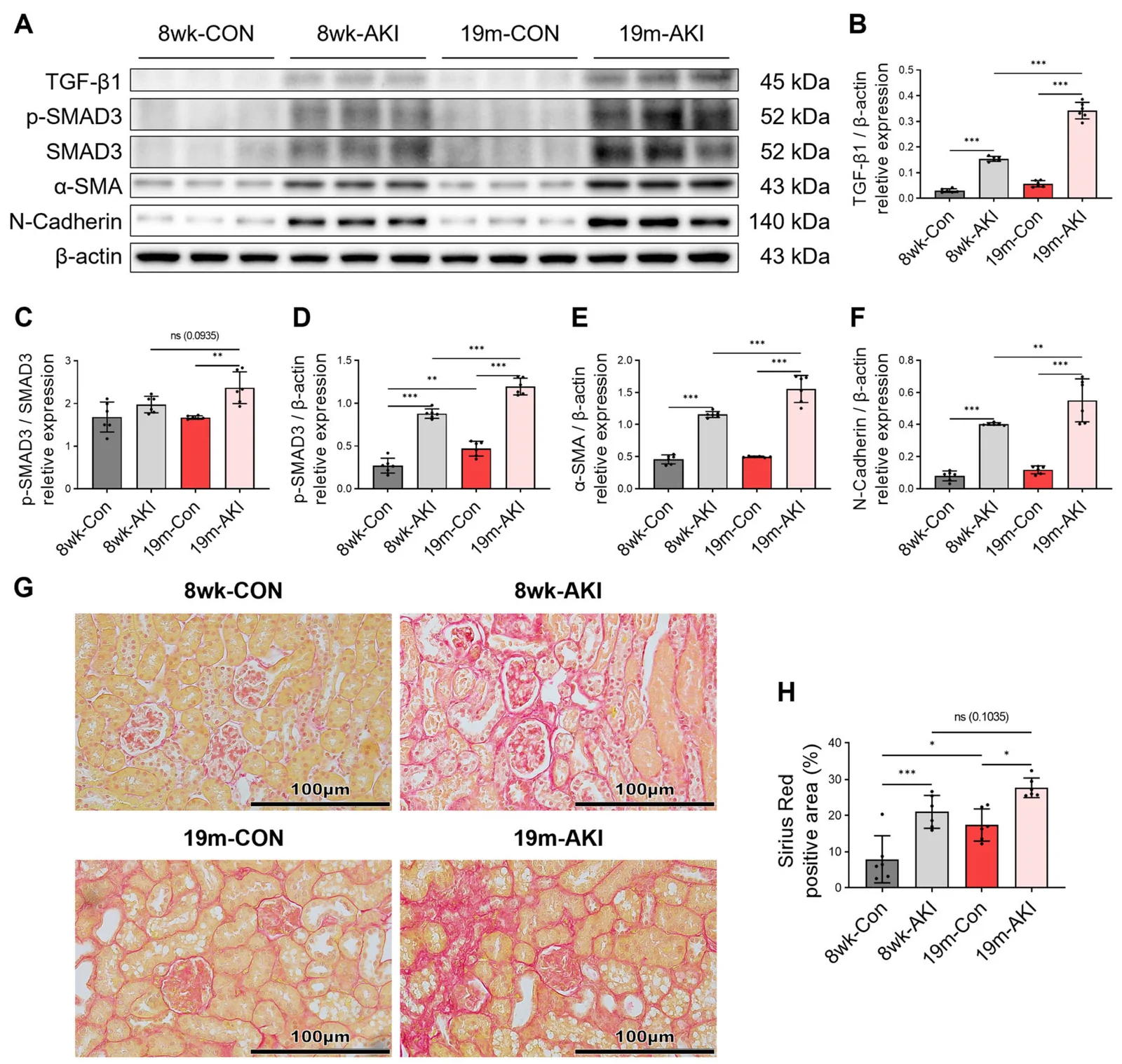
Exacerbation of renal fibrotic remodeling in aged mice after I/R injury. (**A**) Representative Western blot analysis of pro-TGF-β1, p-SMAD3, SMAD3, α-SMA and N-Cadherin expression in mouse kidneys, and (**B**–**F**) their quantification normalized to β-actin or total SMAD3 (*n* = 6 per group). (**G**) Representative Sirius Red staining in the kidney sections. Scale bar = 100 µm (400×). (**H**) Quantification of Sirius Red-positive area (%) (*n* = 6 mice per group). Data are expressed as mean ± SD. Statistical significance was analyzed using one-way ANOVA followed by Tukey’s post hoc test (*** *p* < 0.001, ** *p* < 0.01, and * *p* < 0.05).

To determine whether renal fibrosis contributed to the exacerbation of I/R-induced kidney injury in aged mice, fibrosis-associated markers were analyzed. Western blot analysis demonstrated that I/R injury upregulated fibrosis-associated markers in young mice, and this response was significantly increased in aged mice (Figure 2A–F). Specifically, protein levels of pro-TGF-β1, p-SMAD3, alpha-smooth muscle actin (α-SMA), and N-cadherin were markedly higher in the 19m-AKI group than in the 8wk-AKI group, whereas the p-SMAD3/SMAD3 ratio showed an increasing tendency (*p*-value = 0.0935). Sirius Red staining showed numerically greater collagen-positive areas in the 19m-AKI group than in the 8wk-AKI group, although the difference did not reach statistical significance (*p*-value = 0.1035) (Figure 2G,H). The fibrotic area substantially expanded in aged kidneys subjected to ischemic insult, with prominent interstitial collagen deposition and tubulointerstitial remodeling. Collectively, these results suggest that enhanced renal fibrosis underlies the aggravated kidney injury observed in aged mice following I/R-induced kidney injury, contributing to sustained renal dysfunction and tissue remodeling.

### 3.3. Expression of Apelinergic Components Is Altered in Aged Kidneys Following I/R Injury

**Figure 3:**
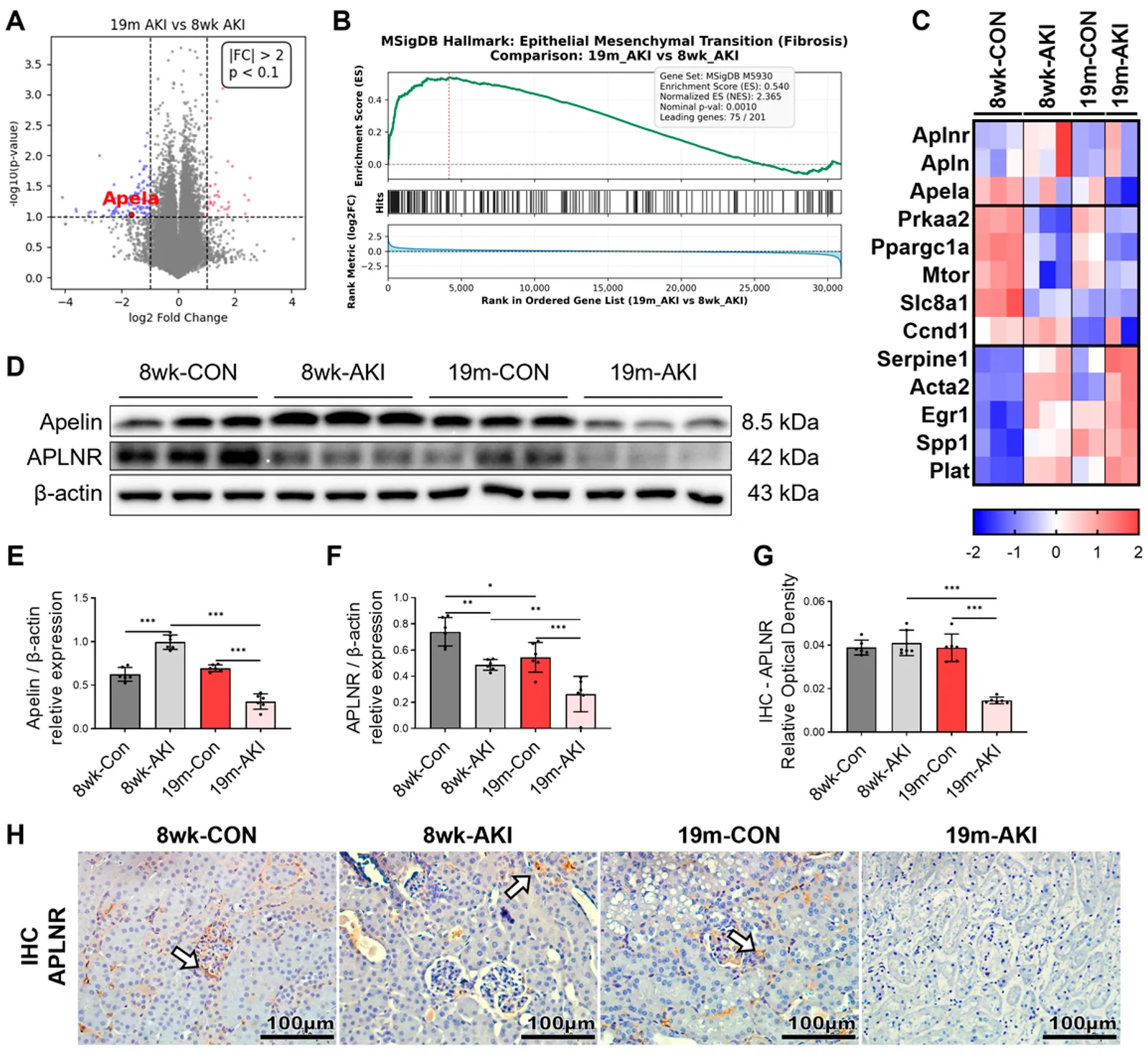
Altered expression of apelinergic components in aged mice following I/R injury. (**A**) Volcano plot highlighting the differentially expressed genes, including *Apela*, between 19m-AKI and 8wk-AKI groups (|FC| > 2, raw *p* < 0.1). (**B**) Pre-ranked GSEA running plot of the MSigDB Hallmark Epithelial–Mesenchymal Transition gene set (M5930) comparing 19m-AKI to 8wk-AKI (ES = 0.540, NES = 2.365, nominal *p* = 0.0010). (**C**) Heatmap showing individual-sample row Z-scores of 13 genes across all four groups (8wk-CON, 8wk-AKI, 19m-CON, and 19m-AKI), encompassing core apelinergic components (*Aplnr*, *Apela*, *Apln*) alongside their downstream signaling effectors and regulated target genes. (**D**) Representative Western blot analysis of apelin and apelin receptor (APLNR) expression in mouse kidneys, and (**E**,**F**) their quantification normalized to β-actin (*n* = 6 per group). (**G**) Quantitative analysis of immunohistochemical staining for APLNR (*n* = 6 mice per group). (**H**) Representative immunohistochemical staining for APLNR in kidney sections (positive signals indicated by arrows). Scale bar = 100 µm (400×). Data are expressed as mean ± SD. Statistical significance was analyzed using one-way ANOVA followed by Tukey’s post hoc test (*** *p* < 0.001, ** *p* < 0.01, and * *p* < 0.05).

To explore transcriptional differences between young and aged kidneys following renal I/R injury, transcriptomic profiling was performed and visualized using volcano plots. The analysis revealed lower *Apela* expression in the 19m-AKI group than in the 8wk-AKI group. In contrast, *Apln* and *Aplnr* did not show statistically significant differences between the 8wk-AKI and 19m-AKI groups in the microarray analysis (Figure 3A). To further characterize transcriptional changes associated with renal fibrotic remodeling in aged mice following I/R injury, pre-ranked GSEA was performed against the standardized MSigDB Hallmark collection. GSEA demonstrated significant positive enrichment of the Hallmark EMT/pro-fibrotic gene set in the 19m-AKI group compared to the 8wk-AKI group (ES = 0.540, NES = 2.365, nominal *p* = 0.0010; Figure 3B), consistent with enhanced expression of genes associated with fibrotic remodeling in aged kidneys following I/R injury.

To further explore the expression patterns of the apelinergic axis and associated pathways across aging and ischemic conditions, a row-wise Z-score heatmap of 13 related genes was generated across all four experimental groups (Figure 3C). First, regarding the apelinergic components (*Aplnr*, *Apln*, and *Apela*), the heatmap showed distinct expression patterns across the four experimental groups. *Aplnr* and *Apln* showed relatively higher expression in the 8wk-AKI group than in the 8wk-CON group, whereas this pattern was less evident in aged mice. *Apela* expression was relatively low in the 19m-AKI group, consistent with the significant difference identified in the direct comparison between the 8wk-AKI and 19m-AKI groups. Second, genes related to mitochondrial and metabolic homeostasis (*Prkaa2*, *Ppargc1a*, *Mtor*, *Slc8a1*, and *Ccnd1*) showed relatively higher expression patterns in the 8wk-CON group and generally lower expression patterns in the aged and I/R-injured groups. Third, pro-fibrotic and senescence-associated genes (*Serpine1*, *Acta2*, *Egr1*, *Spp1*, and *Plat*) tended to show relatively higher expression in the 19m-AKI group.

We next examined Apelin and APLNR expression at the protein level. I/R injury significantly increased Apelin protein expression in young mice (8wk-CON vs. 8wk-AKI), whereas this increase was not observed in aged mice, with lower Apelin protein expression in the 19m-AKI group than in the 8wk-AKI group (Figure 3D,E). Furthermore, APLNR expression was lower in aged mice and lowest in the 19m-AKI group (Figure 3D,F). Immunohistochemical staining supported this result, as the APLNR-positive area was markedly lower in the 19m-AKI group compared with the other three groups (Figure 3G,H). Together, these findings suggest that the apelinergic system is altered in aged kidneys following I/R injury, characterized by reduced Apelin and APLNR protein expression and differential expression of *Apela* under I/R conditions.

### 3.4. Synthetic Apelin-13 Pretreatment Attenuates I/R-Induced Renal Injury in Aged Mice

**Figure 4:**
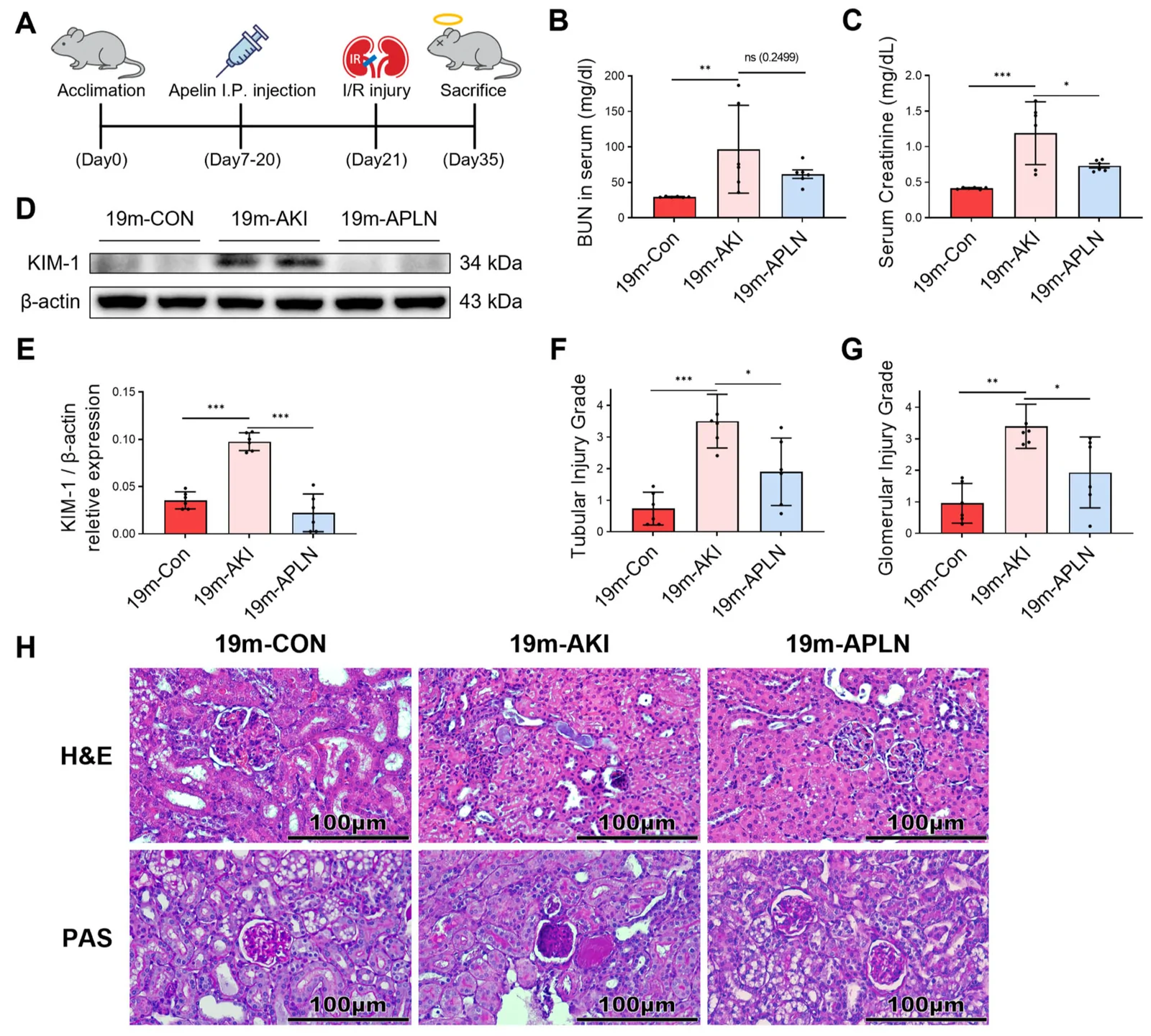
Preventive effects of Apelin against I/R-induced renal injury in aged mice. (**A**) Schematic timeline of the apelin-treated unilateral renal I/R model. (**B**) Serum BUN levels in each group (*n* = 6 mice per group). (**C**) SCr levels in each group (*n* = 6 per group). (**D**) Representative Western blot analysis of KIM-1 expression in mouse kidneys and (**E**) its quantification normalized to β-actin (*n* = 6 per group). (**F**) Evaluation of tubular injury grade and (**G**) glomerular injury grade (*n* = 6 mice per group). (**H**) Representative histological images of kidney sections stained with H&E (top row) and PAS (bottom row). Scale bar = 100 µm (400×). Data are expressed as mean ± SD. Statistical significance was analyzed using one-way ANOVA followed by Tukey’s post hoc test (*** *p* < 0.001, ** *p* < 0.01, and * *p* < 0.05).

To evaluate the protective potential of synthetic Apelin-13 pretreatment in attenuating renal damage in aged mice, synthetic Apelin-13 was administered to 19m mice prior to I/R-induced kidney injury (Figure 4A). BUN levels were numerically lower in the 19m-APLN group than in the 19m-AKI group, although the difference was not statistically significant (*p*-value = 0.2499), whereas SCr levels were significantly decreased (Figure 4B,C). Protein expression of KIM-1 was also significantly lower in apelin-treated mice (Figure 4D,E). Histological evaluation showed that apelin administration significantly lowered tubular and glomerular injury grade and the representative images show alleviated pathological alterations, demonstrating reduced tubular damage and preserved brush border integrity in the 19m-APLN group (Figure 4F–H). Collectively, these findings suggest that exogenous apelin administration mitigates I/R-induced renal injury in aged mice.

### 3.5. Apelin-13 Pretreatment Attenuates Renal Fibrotic Remodeling in Aged Mice and Is Associated with Reduced pro-TGF-β1 Expression and SMAD3 Phosphorylation

**Figure 5:**
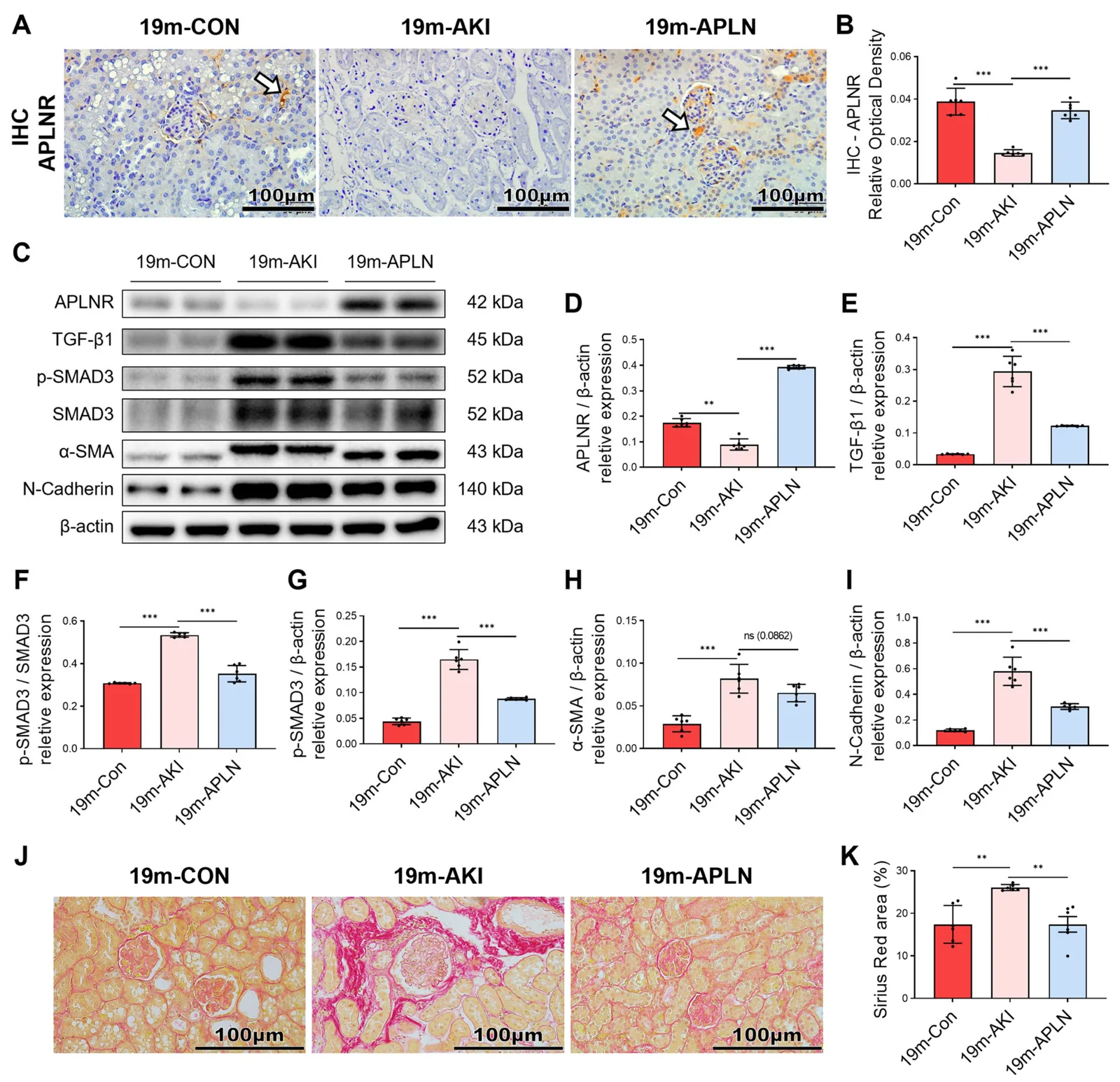
Apelin-13 pretreatment attenuates renal fibrosis in aged mice in association with the downregulation of TGF-β1/SMAD3 signaling. (**A**) Representative immunohistochemical staining for APLNR in kidney sections (positive signals indicated by arrows). Scale bar = 100 µm (400×). (**B**) Quantitative analysis of immunohistochemical staining for APLNR (*n* = 6 mice per group). (**C**) Representative Western blot analysis of APLNR, pro-TGF-β1, p-SMAD3, SMAD3, α-SMA and N-Cadherin expression in mouse kidneys, and (**D**–**I**) their quantification normalized to β-actin or total SMAD3 (*n* = 6 per group). (**J**) Representative Sirius Red staining in the kidney. Scale bar = 100 µm (400×). (**K**) Quantification of Sirius Red-positive area (%) (*n* = 6 mice per group). Data are expressed as mean ± SD. Statistical significance was analyzed using one-way ANOVA followed by Tukey’s post hoc test (*** *p* < 0.001, ** *p* < 0.01).

To investigate whether apelin supplementation modulates renal fibrosis in aged mice, we assessed the expression of fibrosis-related markers in the 19m-APLN group. Immunohistochemistry demonstrated a substantial increase in APLNR-positive areas in the 19m-APLN group compared with the 19m-AKI and 19m-CON groups (Figure 5A,B). Consistently, Western blot analysis confirmed that apelin pretreatment significantly restored APLNR expression compared with the 19m-AKI group (Figure 5C,D). Furthermore, the marked suppression of pro-TGF-β1 expression in the 19m-APLN group was accompanied by significant reductions in SMAD3 phosphorylation (p-SMAD3/SMAD3 ratio), p-SMAD3 levels and N-cadherin expression, along with a decreasing trend in α-SMA expression (*p*-value = 0.0862) (Figure 5C,E–I). Consistent with these changes, Sirius Red staining revealed significantly lower collagen deposition and fibrotic area in the 19m-APLN group compared with the 19m-AKI group (Figure 5J,K). Collectively, these findings demonstrate that Apelin-13 pretreatment attenuated renal fibrotic remodeling in aged mice following I/R injury, accompanied by reduced pro-TGF-β1 expression and SMAD3 phosphorylation.

## 4. Discussion

This study demonstrated that aging increases susceptibility to renal injury and fibrotic remodeling following I/R injury and identified Apelin-13 as a potential modulator of these responses in aged mice. Compared with young mice, aged mice exhibited greater renal functional impairment, histological injury, and fibrotic remodeling following I/R. These changes were accompanied by altered expression of components of the apelinergic system, including reduced Apelin and APLNR protein expression in aged kidneys following I/R injury. Furthermore, prophylactic Apelin-13 administration attenuated renal injury and fibrotic remodeling in aged mice and was associated with reduced pro-TGF-β1 expression and SMAD3 phosphorylation. Together, these findings suggest an association between alterations in apelinergic components and the increased susceptibility of aged kidneys to I/R injury, although the present study does not establish a direct APLNR-dependent mechanism.

Previous studies have established that aged kidneys are more susceptible to acute and chronic injury due to their impaired regenerative capacity, heightened inflammatory responses, and altered hemodynamics [31,32,33]. In the present study, aged mice subjected to I/R exhibited greater increases in BUN, SCr, and KIM-1, together with more extensive tubular injury, than young mice subjected to the same injury. Although the contralateral kidney remained intact in our unilateral I/R model, its compensatory capacity may be reduced with aging. Previous studies using unilateral renal I/R have shown that circulating renal function markers may remain relatively preserved in young mice because of compensation by the contralateral kidney, whereas aged mice exhibit increased plasma urea following unilateral I/R, consistent with an age-associated reduction in renal functional reserve [34]. Thus, the greater increases in BUN and SCr observed in aged mice in the present study may reflect both the greater susceptibility of the ischemic kidney to injury and the diminished compensatory capacity of the contralateral kidney. These findings are consistent with previous evidence that aging impairs renal recovery following AKI and increases susceptibility to maladaptive post-injury remodeling [35].

Renal fibrosis, characterized by the excessive deposition of extracellular matrix (ECM), is a hallmark of maladaptive repair following acute kidney injury [36]. TGF-β1/SMAD3 signaling is a major regulator of renal fibrogenesis, and α-SMA is widely used as a marker of active myofibroblasts [37]. In our study, Sirius Red staining demonstrated prominent interstitial fibrosis following I/R injury (19m-AKI and 8wk-AKI). This fibrotic remodeling was accompanied by increased collagen deposition, upregulation of α-SMA, activation of SMAD3, and elevated N-cadherin expression, all of which were markedly enhanced in aged AKI mice (19m-AKI). In contrast, aged control mice showed substantially less fibrotic remodeling. These observations suggest that aging increases susceptibility to fibrotic responses following ischemic injury rather than being sufficient, under the conditions examined here, to produce a comparable degree of renal fibrosis in the absence of I/R injury.

Transcriptomic analysis provided additional evidence of differences between young and aged kidneys following I/R injury. *Apela* expression was significantly lower in the 19m-AKI group than in the 8wk-AKI group, whereas *Apln* and *Aplnr* transcript levels did not differ significantly between these groups. APELA and Apelin are distinct endogenous ligands of APLNR [38], and the observed reduction in *Apela* should therefore not be interpreted as direct evidence of reduced *Apln* expression or as the basis for the subsequent Apelin-13 intervention. Moreover, because the direct transcriptomic comparison was performed between young and aged mice under I/R conditions, the lower *Apela* expression observed in aged mice cannot be attributed specifically to aging independently of I/R injury. Previous studies have reported reduced renal APELA expression following I/R injury, supporting the responsiveness of APELA to ischemic renal injury [39]; however, evidence for an intrinsic age-dependent decline in renal APELA under basal conditions remains limited. Accordingly, the findings regarding Apela in the present study should be regarded as an exploratory transcriptomic observation in the context of renal I/R injury rather than evidence of an aging-specific alteration.

In parallel, GSEA demonstrated significant positive enrichment of the Hallmark Epithelial–Mesenchymal Transition (EMT) gene set in the 19m-AKI group compared with the 8wk-AKI group. Although this gene set contains numerous genes associated with extracellular matrix remodeling and fibrogenesis, enrichment of the Hallmark EMT gene set does not by itself establish a cellular EMT process in vivo. Therefore, together with the increased expression of fibrosis-associated markers observed in aged I/R kidneys, we interpret this result as proof of enhanced expression of genes associated with fibrotic remodeling rather than direct evidence that EMT is a primary mechanism driving fibrosis in this model. Similarly, the four-group heatmap revealed distinct expression patterns among genes related to the apelinergic system, metabolic homeostasis, fibrosis, and cellular senescence across the experimental groups; however, these descriptive patterns should not be interpreted as evidence of specific age-dependent regulatory mechanisms without direct statistical comparisons.

We next evaluated Apelin and APLNR at the protein level independently of the exploratory transcriptomic findings. Apelin protein expression increased following I/R injury in young mice, whereas this response was blunted in aged mice, resulting in lower Apelin levels in the 19m-AKI group than in the 8wk-AKI group. APLNR protein expression was also reduced in aged kidneys following I/R injury, as demonstrated by Western blotting and quantitative immunohistochemical analysis. These protein-level observations were not intended as direct validation of the *Apela*, *Apln*, or *Aplnr* microarray findings and may reflect regulation occurring at transcriptional or post-transcriptional levels. Collectively, these results demonstrate altered expression of apelinergic components in aged kidneys following I/R injury. However, they do not establish that reduced APLNR expression is causally responsible for the greater susceptibility of aged kidneys to I/R injury.

We subsequently examined whether prophylactic Apelin-13 administration could attenuate the increased susceptibility of aged kidneys to I/R injury. Apelin-13 pretreatment significantly reduced SCr and KIM-1 and attenuated histological injury, whereas BUN was numerically lower but did not differ significantly from that of untreated aged I/R mice. Previous studies have also reported anti-fibrotic effects of apelin in experimental models, including reductions in TGF-β1-associated signaling and extracellular matrix accumulation [25,40]. Consistent with these reports, Apelin-13 pretreatment also reduced collagen deposition and attenuated renal injury and fibrotic remodeling in aged kidneys following I/R injury. At the molecular level, this was accompanied by decreased pro-TGF-β1 expression and SMAD3 phosphorylation (both in the p-SMAD3/SMAD3 ratio and total p-SMAD3 levels), alongside downward trends in α-SMA and N-cadherin. Given the central role of TGF-β1/SMAD3 signaling in myofibroblast activation and extracellular matrix accumulation, these changes are consistent with an attenuated pro-fibrotic molecular response following Apelin-13 pretreatment. However, the present data demonstrate an association between these molecular changes and reduced fibrosis rather than a direct causal mechanism. Similarly, although APLNR expression was increased following Apelin-13 pretreatment, the absence of APLNR blockade or genetic loss-of-function experiments prevents us from concluding that the anti-fibrotic effects of Apelin-13 were mediated specifically through APLNR.

Recent studies further indicate that the effects of Apelin-13 in renal I/R injury may depend substantially on experimental context. Pan et al. reported that Apelin-13 administered at 30 μg/kg attenuated renal I/R injury, with effects associated with lysosomal regulation, restoration of autophagic flux, and modulation of macrophage polarization [41]. In contrast, Lopes-Gonçalves et al. used a higher Apelin-13 dose of 200 μg/kg/day in a bilateral renal I/R model and observed no improvement in acute renal functional or tubular injury markers, together with alterations in tubular proliferative and transport responses [42]. Notably, the dose used in the present study (25 μg/kg/day) was comparable to the 30 μg/kg dose used by Pan et al. but substantially lower than the 200 μg/kg/day regimen used by Lopes-Gonçalves et al. In addition to dose, these studies differed in the timing and duration of Apelin-13 administration, animal age, laterality and duration of ischemia, and post-injury observation period. The present study used aged mice subjected to unilateral 40 min renal ischemia, with Apelin-13 administered prophylactically for 2 weeks before I/R and outcomes evaluated 2 weeks after injury. Therefore, our findings should be interpreted specifically in the context of prophylactic Apelin-13 exposure and subsequent post-I/R fibrotic remodeling in aged kidneys rather than as evidence of a universally protective or therapeutic effect of Apelin-13 in established AKI.

Several limitations of the present study should be acknowledged. First, neither pharmacological APLNR blockade nor genetic loss-of-function approaches were performed. Therefore, although Apelin-13 pretreatment was associated with reduced renal injury and fibrotic remodeling, the present findings do not establish that these effects were mediated specifically through APLNR. Further studies using receptor-specific pharmacological inhibition or genetic approaches will be required to determine APLNR dependence and the downstream pathways responsible for the observed effects. Second, the transcriptomic expression patterns of *Apela*, *Apln*, and *Aplnr* were not independently validated using an additional transcript-level assay such as qPCR. These microarray findings should therefore be regarded as exploratory and require further validation. Third, a separate vehicle-matched control group receiving saline according to the same administration schedule as the Apelin-13-treated group was not included. Although all animals received a single administration of sterile physiological saline immediately after surgery as part of the perioperative procedure, this was not equivalent to a vehicle control for the repeated Apelin-13 treatment; therefore, potential effects associated with the repeated administration procedure cannot be completely excluded. Finally, because Apelin-13 was administered before ischemic injury, the present study evaluated a prophylactic or preconditioning effect rather than treatment of established AKI. In addition, the observation period was limited to 2 weeks after I/R injury, which is insufficient to determine long-term progression to CKD. Future studies employing post-injury treatment regimens and longer follow-up periods will therefore be required to establish the therapeutic potential of Apelin-13 and its effects on long-term renal outcomes.

## 5. Conclusions

In conclusion, aging was associated with greater renal injury and fibrotic remodeling following I/R injury, accompanied by altered expression of apelinergic components. Prophylactic Apelin-13 administration attenuated renal injury and interstitial fibrosis in aged mice, with reduced collagen deposition accompanied by lower pro-TGF-β1 expression and SMAD3 phosphorylation. Although the receptor-dependent mechanisms remain to be established, these findings support a potential preventive effect of Apelin-13 against post-I/R renal fibrotic remodeling in aged kidneys.

## Data Availability

The data that support the findings of this study are available on request from the corresponding author. The data are not publicly available due to privacy or ethical restrictions.

## Supplementary Materials

The following supporting information can be downloaded at https://www.mdpi.com/article/10.3390/biomedicines14092033/s1. Table S1: Reagents and antibodies list.

## Abbreviations

The following abbreviations are used in this manuscript:

AKI	Acute Kidney Injury
ANOVA	Analysis of Variance
APLN	Apelin
APLNR	Apelin Receptor
ARRIVE	Animal Research: Reporting of In Vivo Experiments
BCA	Bicinchoninic Acid
BUN	Blood Urea Nitrogen
CKD	Chronic Kidney Disease
DAB	Diaminobenzidine
DEG	Differentially Expressed Genes
ECM	Extracellular Matrix
EMT	Epithelial-Mesenchymal Transition
FC	Fold Change
GSEA	Gene Set Enrichment Analysis
H&E	Hematoxylin and Eosin
I/R	Ischemia/Reperfusion
KIM-1	Kidney Injury Molecule-1
NIH	National Institutes of Health
OD	Optical Density
PAS	Periodic Acid-Schiff
PBS	Phosphate-Buffered Saline
PBS-T	PBS containing 0.1% Tween-20
RIPA	Radio-Immunoprecipitation Assay
RMA	Robust Multi-array Average
SCr	Serum Creatinine
SD	Standard Deviation
SDS	Sodium Dodecyl Sulfate
SMAD	Mothers against decapentaplegic homolog
TGF-β1	Transforming Growth Factor-beta 1
α-SMA	Alpha-Smooth Muscle Actin
