# Supplementary material for: Apelin-13 Pretreatment Attenuates Age-Associated Renal Fibrotic Remodeling Following I/R Injury
Source: Biomedicines. 2026 Sep 10;14(9):2033. doi: 10.3390/biomedicines14092033 (PMC13604551; doi:10.3390/biomedicines14092033)
Supplement: Supplementary file 1 [file biomedicines-14-02033-s001.zip › Supplementary File Table S1.pdf]

Table S1. Reagents and antibodies list

Category	Reagent / Target	Application	Manufacturer	Cat. No.
**Peptide / Agonist**	Apelin-13	Intraperitoneal injection	MedChemExpress	HY-P1944
**Primary Antibody**	anti-APLNR	Immunohistochemistry	Thermo Fisher Scientific	702069
	anti-β-Actin	Western Blot	Abcam	ab8229
	anti-KIM-1	Western Blot	MyBioSource	MBS2006453
	anti-apelin	Western Blot	Boster Bio	PA1501
	anti-APLNR	Western Blot	Santa Cruz Biotechnology	sc-517300
	anti-TGF-β1	Western Blot	Abcam	ab64715
	anti-p-SMAD3	Western Blot	ABclonal	AP0727
	anti-SMAD3	Western Blot	Santa Cruz Biotechnology	sc-101154
	anti-α-SMA	Western Blot	Abcam	ab5694
	anti-N-Cadherin	Western Blot	Cell Signaling Technology	#13116
**Secondary Antibody**	HRP-anti-mouse IgG	Immunohistochemistry, Western Blot	GenDEPOT	SA001
	HRP-anti-rabbit IgG	Immunohistochemistry, Western Blot	GenDEPOT	SA002
